# Rescue of perfluorooctanesulfonate (PFOS)-mediated Sertoli cell injury by overexpression of gap junction protein connexin 43

**DOI:** 10.1038/srep29667

**Published:** 2016-07-20

**Authors:** Nan Li, Dolores D. Mruk, Haiqi Chen, Chris K. C. Wong, Will M. Lee, C. Yan Cheng

**Affiliations:** 1 The Mary M. Wohlford Laboratory for Male Contraceptive Research, Center for Biomedical Research, Population Council, 1230 York Ave, New York 10065, New York, USA; 2 Department of Biology, Hong Kong Baptist University, Hong Kong, China; 3 School of Biological Sciences, The University of Hong Kong, Hong Kong, China

## Abstract

Perfluorooctanesulfonate (PFOS) is an environmental toxicant used in developing countries, including China, as a stain repellent for clothing, carpets and draperies, but it has been banned in the U.S. and Canada since the late 2000s. PFOS perturbed the Sertoli cell tight junction (TJ)-permeability barrier, causing disruption of actin microfilaments in cell cytosol, perturbing the localization of cell junction proteins (e.g., occluden-ZO-1, N-cadherin-ß-catenin). These changes destabilized Sertoli cell blood-testis barrier (BTB) integrity. These findings suggest that human exposure to PFOS might induce BTB dysfunction and infertility. Interestingly, PFOS-induced Sertoli cell injury associated with a down-regulation of the gap junction (GJ) protein connexin43 (Cx43). We next investigated if overexpression of Cx43 in Sertoli cells could rescue the PFOS-induced cell injury. Indeed, overexpression of Cx43 in Sertoli cells with an established TJ-barrier blocked the disruption in PFOS-induced GJ-intercellular communication, resulting in the re-organization of actin microfilaments, which rendered them similar to those in control cells. Furthermore, cell adhesion proteins that utilized F-actin for attachment became properly distributed at the cell-cell interface, resealing the disrupted TJ-barrier. In summary, Cx43 is a good target that might be used to manage PFOS-induced reproductive dysfunction.

The physiological significance of gap junctions (GJs) to support various cellular functions in mammalian cells and tissues has been well established (for reviews, see refs 1 and 2) including the testis (for reviews, see refs 3, 4, 5, 6). While the testis is known to express more than ten different connexins for the construction of GJ-based intercellular communication channels^7^,^8^, studies have shown that connexin 43 (Cx43)-based gap junctions (GJ)^9^ play important and unique physiological functions (for a review, see ref. 10), at least in Sertoli cells, which apparently cannot be superseded by other connexins. This notion is supported by studies in which Sertoli cell-specific deletion of Cx43 resulted in infertility in mice wherein spermatogonia failed to differentiate into spermatocytes and enter meiosis I/II^11^. Sertoli cells also fail to become differentiated in the testis during adulthood^11^, and the testis of these mice display defects in the expression of multiple genes based on gene profiling search^12^, as well as mis-localization of proteins at the blood-testis barrier (BTB)^13^. Interestingly, fertility is maintained in mice following specific deletion of Cx43 in germ cells^14^, illustrating Cx26 and Cx45 expressed in these mice can supersede the lost function of Cx43 in germ cells^14^, in contrast to Cx43-specific KO in Sertoli cells which leads to infertility^11^. Taken collectively, these studies using genetic models clearly illustrate the unique importance of Sertoli cell Cx43 in spermatogenesis (for a review, see ref. 3).

Other studies have also illustrated the significance of Cx43 in the maintenance of BTB function in the rat testis^15^, such as BTB homeostasis in particular reassembly of the Sertoli cell tight junction (TJ)-permeability barrier^16^. For instance, cell junctions at the BTB undergo continuous remodeling to support the transport of preleptotene spermatocytes connected in clones across the immunological barrier at stage VIII of the epithelial cycle, cell junction dynamics (disassembly, reassembly, stabilization) must be tightly coordinated and regulated through GJs. In a recent study using an animal model in which rats were exposed to an acute dose of adjudin (1-(2,4-dichlorobenzyl)-1H-indazole-3-carbohydrazide, a potential male contraceptive under development (for reviews, see refs 17 and 18)) to induce irreversible BTB disruption and sterility due to meiotic arrest^19^. Overexpression of Cx43 was shown to be able to re-initiate spermatogenesis by re-booting meiosis I/II in adjudin treated rats. For instance, round spermatids were detected in considerable number of tubules *vs.* adjudin treated rats without Cx43 overexpression in the testis^20^. Detailed analysis of tubules that displayed signs of meiosis in these adjudin treated rats has shown that besides corrective spatiotemporal expression of Cx43 in the seminiferous epithelium similar to normal rat testes, F-actin organization was re-built through proper spatiotemporal expression of actin nucleation protein formin 1 and actin barbed end capping and bundling protein Eps8^20^. These changes thus supported proper localization of TJ- (e.g., occludin, ZO-1) and basal ES (ectoplasmic specialization, a testis-specific anchoring junction (for reviews, see refs 21, 22, 23))- (e.g., N-cadherin, γ-catenin) proteins. These findings are significant because they illustrate that the Cx43-based GJ communication is crucial to multiple cellular events to maintain the homeostasis of the BTB, confirming findings of an earlier report regarding the likely involvement of Cx43 in providing cross-talk between various junctions at the BTB to support spermatogenesis^16^.

PFOS (perfluorooctanesulfonate) is an environmental toxicant with its use in consumer products (e.g., carpets, textiles, paints, leather and paper) being banned in the U.S. and Canada in the late 2000s due to its health risks. It continues to be widely used in China due to its ability to serve as a stain repellent, and thus is widely accepted by consumers (for a review, see ref. 24). It was shown that PFOS exerted its effects in Sertoli cells by perturbing F-actin organization in Sertoli cells, thereby disrupting localization of TJ- and basal ES-proteins at the Sertoli cell-cell interface, and associated with a disruption of the GJ-communication function based on a functional dye-transfer FRAP (fluorescence recovery after photobleaching) assay^25^. These phenotypes are somewhat similar to adjudin-induced aspermatogenesis in the testis *in vivo*^19^,^20^. We thus sought to examine if overexpression of Cx43 could rescue the PFOS-mediated Sertoli cell injury, because if it could, these findings offer the basis for therapeutic management of PFOS-induced testis injury through gene therapy by overexpressing Cx43, such as the use of nanoparticles that contain mammalian expression vector with the full-length Cx43 cDNA^26^,^27^,^28^. Herein, we report findings based on the use of an *in vitro* model, which are the basis of future *in vivo* studies.

## Materials and Methods

### Animals and Antibodies

Sprague-Dawley male pups at 15–18 days of age were purchased from Charles River Laboratories (Kingston, NY) and maintained at the Rockefeller University Comparative Bioscience Center (CBC). Each group of ten pups was supplied with a foster mother during shipment and housing at the Rockefeller University CBC, and they had free access to water and standard rat chow. The use of rats was approved by the Rockefeller University Laboratory Animal Care and Use Committee with Protocol Numbers: 12506 and 15780-H. Rats were euthanized by CO_2_ asphyxiation using slow (20–30%/min) displacement of chamber air with compressed CO_2_ using a regulator approved by the Rockefeller University Laboratory Safety & Environmental Health (LS&EH). All experimental protocols and methods including the use of animals for all the studies reported herein were approved by, and carried out in accordance with the relevant guidelines of the Rockefeller University LS&EH, the Rockefeller University Institutional Biosafety Committee (IBC), and the Rockefeller University Comparative Bioscience Center (CBC), which were detailed in the approved Protocols 12506 and 15780-H. Also, these procedures were also described in detail wherever applicable in the sections below. Antibodies were purchased and applied as listed in Table 1.

### Toxicants

Perfluorooctanesulfonate (PFOS, heptadecafluorooctanesulfonic acid, potassium salt, Mr 538.22) was purchased from Sigma-Aldrich (St. Louis, MO) and dissolved in DMSO at 100 mM so that when the Sertoli cells were treated with 20 μM PFOS, the concentration of DMSO in the culture medium was 0.02% (v/v). The same amount of DMSO was used in controls, including Sertoli cells transfected with the empty pCI-neo mammalian expression vector (Promega).

### Isolation of Sertoli cells and Treatment with PFOS

Sertoli cells were isolated form 20-day-old rat testes and cultured in serum-free chemically defined medium of F12/DMEM supplemented with growth factors, including bovine insulin (10 μg/ml), human transferrin (5 μg/ml), and epidermal growth factor (2.5 ng/ml), as well as bacitracin (5 μg/ml) and gentamicin (20 μg/ml) as earlier described^29^. Sertoli cells from 20-day-old rat testes were used because Sertoli cells at this age are fully differentiated and ceased to divide^30^,^31^, and these cells are also functionally similar to Sertoli cells isolated from adult rat testes^32^,^33^. Furthermore, Sertoli cells isolated from 20-day-old rat testes were >98% pure with negligible contamination of germ, peritubular myoid or Leydig cells^34^. Virtually all contaminating germ cells were lysed via a brief hypotonic treatment using 20 mM Tris, pH 7.4 at 22 ^o^C for 2.5 min at room temperature as described^35^. However, Sertoli cells isolated from adult rat testes using the established BSA gradient approach^36^ had a purity of ~85% even after the hypotonic treatment^32^,^33^. As such, most investigators use Sertoli cells isolated from 20-day-old rat testes for their studies^37^,^38^,^39^,^40^,^41^,^42^. For various experiments, Sertoli cells were seeded on Matrigel (BD Biosciences, San Jose, CA; 1:7 diluted in F12/DMEM medium) coated: (i) 6-well dishes at 0.4 × 10^6^ cells/cm^2^, with each well containing 5 ml of F12/DMEM supplemented with growth factors (for immunoblotting); and (ii) cover glasses (round, 18-mm diameter) placed into 12-well dishes at 0.04 × 10^6^ cells/cm^2^ (for immunofluorescent (IF) analysis of F-actin, actin binding/regulatory proteins such as Arp3 and Eps8), 0.15 *vs.* 0.1 × 10^6^ cells/cm^2^ (for IF analysis of Cx43 *vs.* TJ- and basal ES-proteins), with each well containing 2 ml medium. These cell densities were selected based on pilot experiments to better visualize the corresponding target proteins either in the Sertoli cell cytosol or at the cell-cell interface. For instance, a Sertoli cell density of 0.15 × 10^6^ cells/cm^2^ was necessary to obtain optimal visualization of Cx 43. Cover glasses were then removed from the 12-well dishes and processed for immunofluorescence microscopy. To avoid inter-experimental variations, all cover glasses within an experiment were processed simultaneously. For experiments to assess the Sertoli cell TJ-permeability function to monitor BTB integrity *in vitro*, Sertoli cells at 1.0 × 10^6^ cells/cm^2^ were seeded onto Matrigel-coated (1:5 diluted in F12/DMEM) Millicell HA bicameral units (12 mm diameter, 0.6 cm^2^ effective surface area; 0.45 μm pore size; EMD Millipore), and units were then placed in 24-well dishes with the apical and basal compartment containing 0.5-ml F12/DMEM supplemented with growth factors as described^29^. Each time point had quadruple bicameral units. The time of cell plating onto the bicameral units or cover glasses was defined as day 0. On day 2, cells were subjected to a hypotonic treatment with 20 mM Tris (pH 7.4 at 22 °C) for 2 min to lyse residual germ cells as described^35^. Thereafter cells were washed twice with F12/DMEM, and the purity of these Sertoli cells was >98% when primer pairs specific to markers of Sertoli, Leydig, germ or myoid cells were used for RT-PCR, which confirmed negligible cell contamination as described^43^. On day 3, PFOS dissolved in DMSO was diluted in F12/DMEM supplemented with various growth factors and gentamicin as described^29^ to obtain the desired final concentration of 20 μM. Sertoli cells were exposed to PFOS for 24 hr before termination. For the control, vehicle (i.e., F12/DMEM containing 0.02% DMSO (v/v)) was used.

### Transfection of Sertoli cells with Plasmid DNA for Cx43 Overexpression

The full length rat testicular Cx43 cDNA (GenBank Accession Number BC081842.1) obtained by PCR using Sertoli cell cDNAs with both start and stop codons was cloned into the pCI-neo mammalian expression vector (Promega, Madison, WI) as earlier described^20^. Possible endotoxin contamination in the plasmid DNA was removed using the EndoFree Plasmid Mega Kit (Qiagen, Germantown, MD). The sequence of this Cx43 cDNA clone was confirmed by direct nucleotide sequencing analysis at Genewiz (South Plainfield, NJ). Sertoli cells were transfected with pCI-neo empty vector (Ctrl) *vs.* pCI-neo containing Cx43 plasmid DNA (pCI-neo/Cx43) by using Effectene Transfection Reagent (Qiagen) for 6 hr on day 2. For cultures to be used for immunoblot analysis, Sertoli cells were transfected with 1.6 μg plasmid DNA in 200 μl Buffer EC, 12.8 μl Enhancer, 24 μl Effectene and 2 ml F12/DMEM with supplements and antibiotics. For cell staining and TER measurement, cells were transfected with 0.2 μg plasmid DNA in 25 μl Buffer EC, 1.6 μl Enhancer, 3 μl Effectene and 0.5 ml F12/DMEM with supplements and antibiotics. To monitor successful transfection, plasmid DNA was labeled with Cy3 (red fluorescence) using a *Label* IT Tracker Intracellular Nucleic Acid Localization Kit (Mirus).

### Functional Assay that Monitors Sertoli cell TJ-Permeability Barrier Function

The Sertoli cell TJ-permeability barrier function *in vitro* was monitored by quantifying the transepithelial electrical resistance (TER) across the cell epithelium with a Millicell ERS system (EMD Millipore, Billerica, MA) as described^29^,^44^. In brief, Sertoli cells were seeded on Matrigel (1:5 diluted with F12/DMEM) -coated Millicell HA bicameral units at 1.0 × 10^6^ cells/cm^2^ and cultured for 2 days to allow the assembly of functional TJ-barrier. Sertoli cells were then transfected with plasmid DNA on day 2 for 6 hr. Thereafter, cells were washed twice with F12/DMEM to remove transfection reagents, and they were cultured for an additional 18 hr before cells were exposed to PFOS for 24 hr. TER reading was taken daily using quadruple bicameral units for each treatment group including controls, medium was replaced immediately after TER reading was taken. Each TER experiment was performed three independent times using different batches of Sertoli cells, which yielded similar results.

### Immunoblotting and Immunofluorescent Microscopy (IF)

Sertoli cell lysates were obtained by using immunoprecipitation (IP) lysis buffer [10 mM Tris, pH 7.4 at 22 ^o^C, containing 0.15 M NaCl, 1% NP-40 (v/v), and 10% glycerol (v/v)] freshly supplemented with protease and phosphatase inhibitor cocktails (Sigma-Aldrich) as described^44^. Following homogenization using a Cole-Parmer Ultrasonic Processor, cell lysates were centrifuged at 15,000 *g* for 60 min at 4 ^o^C to remove cellular debris. About 20 μg cell lysate protein was subjected to SDS-PAGE and immunoblotting^44^,^45^ to detect changes in the steady-state levels of selected target proteins, including Cx43 after treatment of PFOS with or without overexpression of Cx43 *vs.* controls. About 10 μg protein of cell lysate was used per lane to visualize ß-actin and vimentin, which also served as the protein loading control. IF using cultured Sertoli cells was performed as described^44^. Primary antibodies used for different experiments in this report are listed in Table 1, with goat anti-mouse or goat anti-rabbit IgG conjugated to Alexa Fluor 488 (Invitrogen) diluted at 1:250 in PBS (10 mM sodium phosphate, pH 7.4 at 22 ^o^C, containing 0.15 M NaCl). Cells were mounted in Prolong Gold Antifade reagent containing DAPI (Invitrogen) to visualize cell nuclei, and images were acquired using MicroSuite FIVE software (Version 1.224, Olympus Soft Imaging Solutions Corp., Lakewood, DO) using an Olympus DP71 12.5 MP digital camera attached to an Olympus BX61 motorized microscope (Olympus America, Inc., Center Valley, PA). Representative micrographs are shown but each experiment was repeated at least three times using different preparations of Sertoli cells, which yielded similar results.

### Real-Time PCR (qPCR)

qPCR was performed as earlier described^20^. In short, Sertoli cells cultured alone for 3 days were treated with PFOS (20 μM) for 24 hr and terminated on day 4. Total RNA was isolated from these Sertoli cells using Trizol (Life Technologies) and reversed transcribed to cDNAs using Moloney murine leukemia virus reverse transcriptase (Promega). qPCR was performed using the QuantStudio^TM^ 12 K Flex Real-time PCR System (Thermo Fisher) at the Rockefeller University Genomics Resource Center (*n *= 3 experiments, each in triplicate) using a primer pair specific to rat Cx43 (sense: 5′-ACTCTCGCCTATGTCTCCT-3′, nucleotides 1023–1041; antisense: 5′-CGAGTTGGAGATGGTGCTT-3′, nucleotides 1164–1182; GenBank Accession Number: BC081842.1), and a primer pair specific to GAPDH for co-amplification as described^20^ which served as the internal control for normalization. The specificity of the fluorescence signal was confirmed by both melting curve analysis and gel electrophoresis. The steady-state level of Cx43 was then determined using the 2^−ΔΔCT^ method.

### GJ Communication Assay by Fluorescent Dye-transfer

A fluorescence-based GJ-intercellular communication assay to assess the effects of overexpression of Cx43 on Sertoli cells treated with PFOS was performed as described^16^,^25^,^46^. Sertoli cells cultured at 0.15 × 10^6^ cells/cm^2^ on Matrigel-coated (diluted 1:7 with F12/DMEM) glass-bottom dishes (Cat #: P35G-0-20-C, MatTek) were transfected with plasmid DNA for overexpression of Cx43 *vs.* empty pCI-neo vector (control) on day 2 for 6 hr, and then exposed to PFOS *vs.* without PFOS (control) on day 3 for 24 hr. Cells were labeled with 5 μM calcein AM (Mr 994.87, Invitrogen) for 30 min at 35 ^o^C. Cell membrane-permeable calcein AM would be convereted in living cells into membrane-impermeable and fluorescent calcein. Extracellular calcein AM was then removed after incubation and cells were rinsed with F12/DMEM twice. A few selected Sertoli cells were photobleached and the FRAP (fluorescence recovery after photobleaching) was performed by monitoring changes in the fluorescent intensity of the selected Sertoli cell that assessed the transfer of fluorescent dye from adjacent Sertoli cells to the photobleached cell. GJ communication assay was performed at the Rockefeller University BioImaging Resource Center using confocal microscopy (Zeiss/Perkin-Elmer) equipped with a Digital Mosaic system (Photonics Instruments) for photobleaching and to acquire images from 0 to 120 sec with Sertoli cells maintained in a culture chamber at 35 ^o^C in a humidified atmosphere of 5% CO_2_/95% air (v/v). Images were acquired using an Andor iXon EMCCD camera and MetaMorph software package (Molecular Devices), and stacked images were aligned using the StackReg plug-in in ImageJ for analysis.

### Image analysis

Image analysis to assess the fluorescent intensity (FI) or fluorescent signal (FS) at the Sertoli cell-cell interface was performed as described^44^. At least 200 cells were randomly selected and examined in control *vs.* experimental groups with *n *= 3 experiments (i.e., ~70 randomly selected cells per experiment). For changes in protein localization near the Sertoli cell surface, the distribution of the fluorescent signals at the cell-cell interface was measured at the two opposite ends (i.e., 4 measurements) of the Sertoli cell nucleus, which was then averaged to obtain the mean width. The fluorescent intensity of a target protein in Sertoli cells was quantified using ImageJ 1.45 software (NIH, Bethesda, MD; http://rsbweb.nih.gov/ij).

### Statistical Analysis

Statistical significance was determined by two-way ANOVA, followed by Dunnett’s procedure using GB-STAT Statistical Analysis Software Package (Version 7.0, Dynamic Microsystems, Silver Spring, MD). All experiments were repeated at least three times using different batches of Sertoli cell cultures, and each time point had triplicate or quadruplicate (for TER measurements that monitored TJ-permeability barrier function) wells/dishes.

## Results

### PFOS (Perfluorooctanesulfonate) Down-Regulates Cx43 expression and Perturbs the Organization of Actin Microfilaments at the Sertoli cell Basal ES (Ectoplasmic Specialization)/BTB (Blood-Testis Barrier)

**Figure 1:**
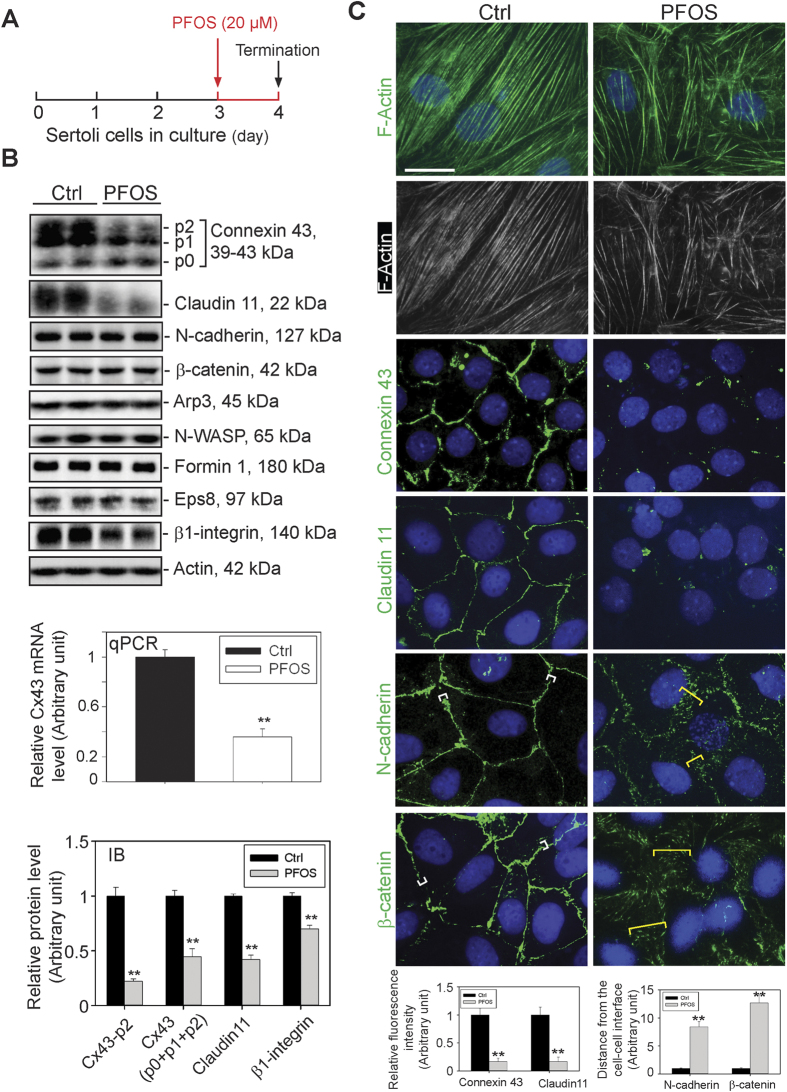
Disruptive effects of PFOS on Sertoli cell F-actin organization and distribution of BTB-associated proteins at the cell-cell interface. (**A**) Treatment regimen used for this experiment. Sertoli cells cultured alone at 0.4 × 10^6^ cell/cm^2^ for 3 days following the establishment of a functional TJ-barrier were treated with 20 μM PFOS for 24 hr. Thereafter, cells were harvested for immunoblotting or for immunofluorescent microscopy. (**B**) Immunoblot analysis to assess changes in the steady-state level of Cx43 (a GJ protein), TJ- (e.g., claudin 11), basal ES (e.g., N-cadherin, ß-catenin), and actin binding/regulatory proteins (e.g., Arp3, N-WASP, formin 1 and Eps8) with ß-actin serving as the protein loading control. Cx43 appeared as three immunoreactive bands designated as p0, p1 and p2, corresponding to Mr of 39, 41, and 43 kDa due to differential phosphorylation, which is consistent with earlier reports^56^,^57^. A study by qPCR (with GAPDH serving as the internal control for normalization) also confirmed a down-regulation of Cx43 expression. Immunoblot analysis also illustrated a down-regulation on the steady-state level of Cx43 protein when p0, p1 and p2 isoforms were analyzed as a whole, with a more considerable reduction for p2. Besides Cx43, claudin 11 and ß1-integrin were also down-regulated. Each bar in the two bar graphs is a mean ± SD of *n* = 5 experiments. **P < 0.01. (**C**) F-actin organization in Sertoli cells was assessed by phalloidin-FITC staining, illustrating PFOS induced truncation and defragmentation of actin microfilaments. PFOS treatment also considerably down-regulated the expression of Cx43 and claudin 11 at the Sertoli-Sertoli cell interface, consistent with data shown in (**B**). While the levels of N-cadherin and ß-catenin were not considerably affected based on immunoblotting results, these basal ES proteins localized diffusely at the Sertoli cell-cell interface, apparently rapidly internalized. Different cell densities were used to optimize the visualization of different target proteins including F-actin as noted in *Materials and Methods*. Micrographs shown herein are representative results of an experiment. All three independent experiments yielded similar results. Scale bar, 25 μm, which applies to all other micrographs. Each bar graph is a mean ± SD of *n* = 3 experiments. **P < 0.01.

The BTB, unlike other blood-tissue barriers such as the blood-brain barrier which is constituted exclusively by the TJ-barrier of the microvessels with support from pericytes in the brain (for a review, see ref. 47), is created by coexisting TJ, basal ES and GJ between adjacent Sertoli cells close to the base of the seminiferous tubule (for reviews, see refs 48, 49, 50). The most typical feature of the basal ES/BTB is the network of actin microfilaments that are extensively bundled that lie perpendicular to the Sertoli cell plasma membrane and sandwiched in between cisternae of endoplasmic reticulum and the Sertoli cell plasma membrane (for reviews, see refs 48 and 51), illustrating the significance of the Sertoli cell F-actin network in supporting BTB function. Studies have shown that Sertoli cells cultured *in vitro* establish a functional TJ-barrier that mimics the Sertoli BTB *in vivo*, which have been used extensively in the field by investigators as a model to study BTB function for almost three decades^40^,^41^,^42^,^52^,^53^,^54^,^55^. Exposure of Sertoli cells cultured *in vitro* with an established functional TJ-permeability barrier to PFOS at 20 μM (a concentration selected based on an earlier dosing study which yielded a distinctive phenotype on the Sertoli cell TJ-barrier function without unwanted cytotoxicity^25^) using the regimen summarized in Fig. 1A, PFOS was found to down-regulate Cx43 expression considerably, in particular the Cx43 p2 isoform of Mr 43 kDa, to be followed by the p1 isoform of Mr 41 kDa, but a mild up-regulation of p0 isoform (Mr 39 kDa) (Fig. 1B). Our findings that Cx43 is a heterogeneous protein, displaying apparent Mr of 39, 41 and 43 kDa corresponding to p0, p1 and p2 (Fig. 1B) are consistent with earlier reports^56^,^57^. This down-regulation of Cx43 by PFOS was also supported by findings using qPCR, illustrating a considerable reduction in its steady-state mRNA level, consistent with immunoblotting data shown in Fig. 1B. For instance, a considerable down-regulation on the steady-state protein of not just p2, but Cx43 as a whole was noted. In short, the expression of Cx43 was down-regulated by PFOS, not just its phosphorylation status. PFOS also down-regulated claudin 11 considerably, apparently a less phosphorylated form due to a reduced Mr (Fig. 1B). However, PFOS did not alter the expression of several other BTB-associated proteins, including actin binding and regulatory proteins Arp3 (an actin barbed end nucleation protein causing actin filament branching), N-WASP (an upstream activator of the Arp2/3 complex), formin 1 (an actin nucleation protein inducing long stretches of actin microfilaments) and Eps8 (an actin barbed end capping and bundling protein), except ß1-integrin which is a hemidesmosome protein^58^ was found to be down-regulated (Fig. 1B). More important, the organization of actin microfilaments in Sertoli cells was found to be grossly affected in which defragmentation was extensively found in the cell cytosol in which actin microfilaments were truncated, appearing as shorter stretches of fragments (see both the green fluorescence and black-and-white images in Fig. 1C). Truncation of actin microfilaments in Sertoli cells failed to support localization of GJ protein Cx43, TJ protein claudin 11 and also basal ES proteins N-cadherin and ß-catenin so that these adhesion proteins no longer tightly localized at the Sertoli cell-cell interface (Fig. 1C).

### Overexpression of Cx43 in Sertoli cells Promotes TJ-Barrier Function, Rescuing PFOS-Induced BTB Disruption

**Figure 2:**
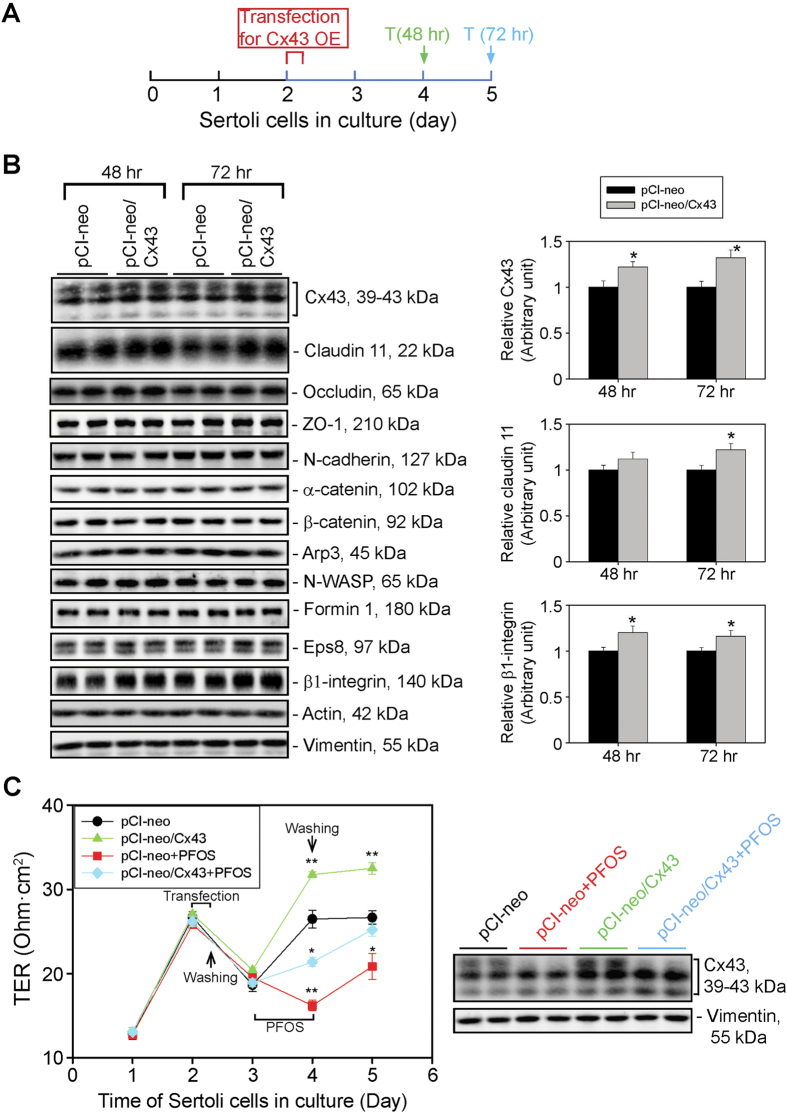
Overexpression of Cx43 in Sertoli cells with an established TJ-permeability barrier promotes barrier function, rescuing PFOS-induced barrier disruption. (**A**) Treatment regimen used to assess the effects of overexpression of Cx43 in Sertoli cells on the steady-state levels of BTB-associated proteins by immunoblot analysis. T, termination. (**B**) Immunoblot analysis using Sertoli cell lysates to assess changes in the steady-state levels of selected BTB-associated proteins, including Cx43 following its overexpression at 48- and 72-hr after transfection (i.e., on days 4 and 5, respectively). Among all the BTB-associated proteins examined, only the TJ protein claudin 11 and the hemidesmosome protein ß1-integrin displayed a moderate up-regulation following Cx43 overexpression as illustrated in the bar graphs on the right panel. Each bar in the bar graphs is a mean ± SD of *n* = 3 experiments. *P < 0.05. (**C**) Sertoli cells were cultured on Matrigel-coated bicameral units at 1.0 × 10^6^ cells/cm^2^ for two days to establish a functional TJ-permeability barrier. Cells were then transfected with pCI-neo empty vector *vs.* pCI-neo vector containing Cx43 for overexpression of Cx43 (pCI-neo/Cx43) in these cells for 6 hr. Thereafter, cells were washed twice with F12/DMEM and cultured for another 18 hr, followed by an incubation with or without (i.e., equal amount of DMSO-containing medium) 20 μM PFOS for 24 hr. Cells were then rinsed twice to remove PFOS and cultured in F12/DMEM for another 24 hr. TER was recorded daily using quadruple bicameral units for each time-point. Overexpression of Cx43 in Sertoli cells was found to promote the TJ-permeability barrier and alleviate PFOS-induced TJ-disruption. Each data point is a mean ± SD of quadruple bicameral units of a representative experiment from *n* = 3 experiments, which yielded similar results. *P < 0.05; **P < 0.01. Sertoli cell lysates obtained on day 5 (see left panel, and 2A for the treatment regimen) were also used for immunoblotting, illustrating overexpression of Cx43 partially rescued the PFOS-induced Cx43 down-regulation. Vimentin served as the protein loading control.

Studies have shown that GJ-based Cx43 is crucial to confer BTB function in the rat testis by mediating cross-talk between various types of junctions at the BTB, such as TJ and basal ES, in order to maintain BTB homeostasis^15^. Additionally, the expression of Cx43 is essential to confer TJ reassembly during BTB remodeling^16^, such as during the transport of preleptotene spermatocytes across the immunological barrier. A recent study also reported that overexpression of Cx43 rescued toxicant-induced BTB disruption by resealing the disrupted BTB, re-initiating meiosis using the adjudin model^20^ since an acute dose of adjudin was shown to cause meiotic arrest^19^. Using the regimen shown in Fig. 2A, overexpression of Cx43 in Sertoli cells was confirmed by immunoblotting (Fig. 2B). Interestingly, TJ protein claudin 11 expression was also moderately up-regulated, and also hemidesmosome protein ß1-integrin but not other BTB-associated proteins examined following overexpression of Cx43 (Fig. 2B). These findings were also summarized and shown in the bar graphs on the right panel (Fig. 2B). It was noted that the efficiency of overexpression of Cx43 using primary Sertoli cells cultured *in vitro* was ~25–35% as noted in the bar graph shown in Fig. 2B (upper panel) when cells were harvested on days 4 and 5 at 48 and 72 hr following the transfection of Sertoli cells with plasmid DNA. This relatively low efficiency may be the result of timing to terminate the cells. Nonetheless, overexpression of Cx43 was found to promote the Sertoli cell TJ-permeability barrier (Fig. 2C). Furthermore, Cx43 overexpression also rescued PFOS-induced Sertoli cell TJ-permeability barrier disruption (Fig. 2C). These findings were also corroborated by quantifying changes in the Cx43 steady-state using Sertoli cell lysates obtained on day 5 in the four experimental groups, illustrating overexpression of Cx43 partially restored the PFOS-induced Cx43 down-regulation (Fig. 2C, right panel).

### Overexpression of Cx43 recues the PFOS-mediated GJ communication dysfunction

**Figure 3:**
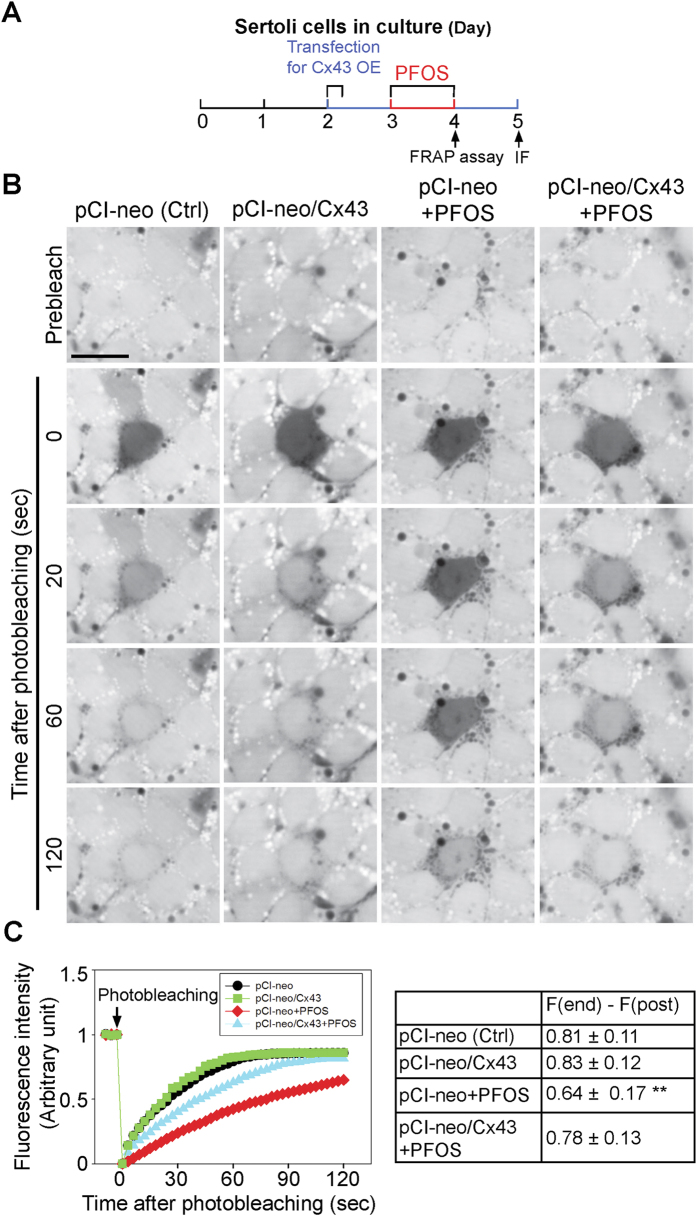
Overexpression of Cx43 in Sertoli cells rescues PFOS-induced GJ communication dysfunction. (**A**) Treatment regimen used to determine if overexpression (OE) of Cx43 can rescue PFOS-induced GJ communication function based on a dye-transfer FRAP (fluorescence recovery after photobleaching) functional assay. (**B**) GJ intercellular communication function between Sertoli cells cultured at 0.15 × 10^6^ cells/cm^2^ was assessed by a dye-transfer FRAP assay based on the transfer of fluorescent dye (calcein AM) from neighboring cells via GJ following photobleaching of a single Sertoli cell. Sertoli cells were cultured at 0.15 × 10^6^ cells/cm^2^ on Matrigel-coated glass-bottom dishes for 2 days. The cells were then transfected with pCI-neo/Cx43 *vs.* pCI-neo empty vector for 6 hr. Thereafter, cells were washed twice with F12-DMEM and cultured for another 18 hr, followed by treatment with 20 μM PFOS for 24 hr (with or without Cx43 overexpression vs. Cx43 overexpression alone or empty vector alone). Results from a typical FRAP assay are shown with *n* = 3 experiments, which yielded similar results. Scale bar, 40 μm, which applies to all other micrographs. **(C)** Typical results of a representative FRAP assay from *n *= 3 experiments that yielded similar results, illustrating GJ communication was impaired by PFOS treatment, but overexpression of Cx43 rescued the PFOS-induced GJ dysfunction. Each data point on the right panel is a mean ± SD of *n* = 4 independent experiments for the four experimental groups using different batches of Sertoli cells for the FRAP assay. **P < 0.01.

Using the regimen shown in Fig. 3A, PFOS was found to impede Sertoli cell GJ communication function based on the use of a dye-transfer functional assay by quantifying the ability of GJs between Sertoli cells to transfer fluorescent dye across the GJ to a photobleached cell (Fig. 3B) in a FRAP assay. Importantly, overexpression of Cx43 was found to rescue the PFOS-induced GJ-based intercellular communication dysfunction (Fig. 3B), and also supported by findings of fluorescent dye transfer assay of *n *= 3 experiments (Fig. 3C), illustrating Cx43 plays a critical role in GJ communication function in the testis.

### Overexpression of Cx43 re-organizes TJ- and basal ES-protein distribution at the Sertoli cell-cell interface disrupted by PFOS

**Figure 4:**
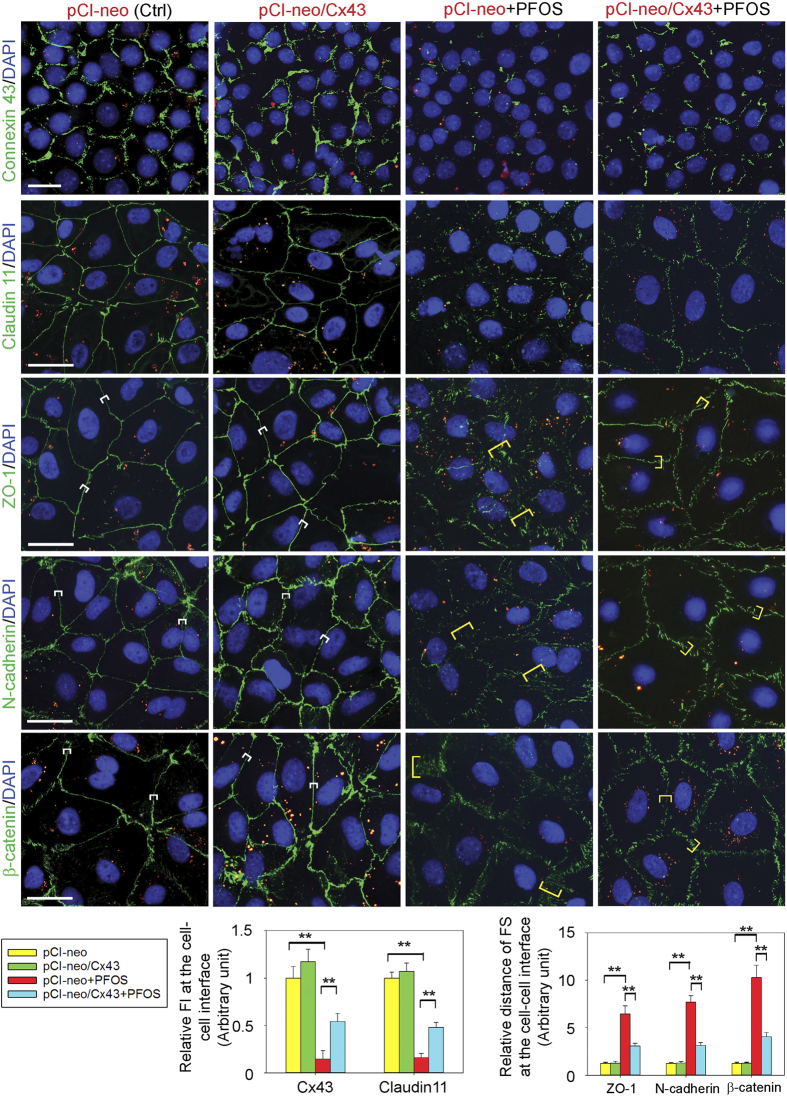
Overexpression of Cx43 in Sertoli cells rescues PFOS-induced Sertoli cell BTB disruption through proper localization of adhesion proteins at the cell-cell interface. Sertoli cells were cultured at 0.1–0.15 × 10^6^ cells/cm^2^ for two days. They were transfected with pCI-neo/Cx43 *vs.* pCI-neo empty vector (control, Ctrl) and then subjected to PFOS treatment, and cells were harvested for IF analysis as noted in the regimen shown in Fig. 3A. In cells overexpressed with Cx43 (pCI-neo/Cx43), the fluorescent intensity for Cx43 at cell-cell interface was moderately stronger than control (pCI-neo empty vector alone) cells. However, PFOS induced considerable down-regulation of Cx43 and claudin 11 at the cell-cell interface (pCI-neo+PFOS), PFOS also perturbed the localization of TJ protein ZO-1, and basal ES proteins N-cadherin and β-catenin. Importantly, overexpression of Cx43 in PFOS treated cells (pCI-neo/Cx43+PFOS) rescued the PFOS-mediated BTB disruption by maintaining the expression of Cx43 and claudin 11. This also induced proper localization of TJ protein ZO-1 and basal ES proteins N-cadherin and ß-catenin at the cell-cell interface. Plasmid DNA (pCI-neo vs. pCI-neo/Cx43) was labeled with Cy3 and appeared as red fluorescence to annotate successful transfection. Scale bar, 30 μm, in the first micrograph, which applies to the remaining micrographs in the same panel. These results are representative micrographs from an experiment, and *n* = 3 independent experiments were performed using different batches of Sertoli cells and yielded similar results. Each bar graph is a mean ± SD of *n* = 3 experiments. **P < 0.01. FI, fluorescent intensity; FS, fluorescent signal.

Following exposure of Sertoli cell epithelium *in vitro* to PFOS, the localization of Cx43, TJ- (e.g., claudin 11, ZO-1) and basal ES- (e.g., N-cadherin, ß-catenin) proteins at the Sertoli cell-cell interface was found to be grossly perturbed, either considerably down-regulated (e.g., Cx43, claudin 11) or mis-localized (e.g., ZO-1, N-cadherin, ß-catenin) (Fig. 4). Overexpression of Cx43 moderately promoted localization of Cx43, as well as TJ and basal ES proteins at the Sertoli cell-cell interface (Fig. 4). More important, overexpression of Cx43 in Sertoli cells before these cells were exposed to PFOS was found to rescue these cells from the disruptive effects of PFOS. For instance, Cx43 overexpressed Sertoli cells displayed proper expression and/or localization of BTB-associated proteins at the Sertoli cell-cell interface exposed to PFOS (Fig. 4), making them more similar to control cells transfected with empty vector (Fig. 4). These findings thus support earlier observations that overexpression of Cx43 rescued the PFOS-induced Sertoli cell TJ-permeability barrier disruption as noted in Fig. 2C.

### Overexpression of Cx43 Induces reorganization of Actin Microfilaments in Sertoli Cells Exposed to PFOS Through Proper Spatiotemporal Expression of Arp3 and Eps8

**Figure 5:**
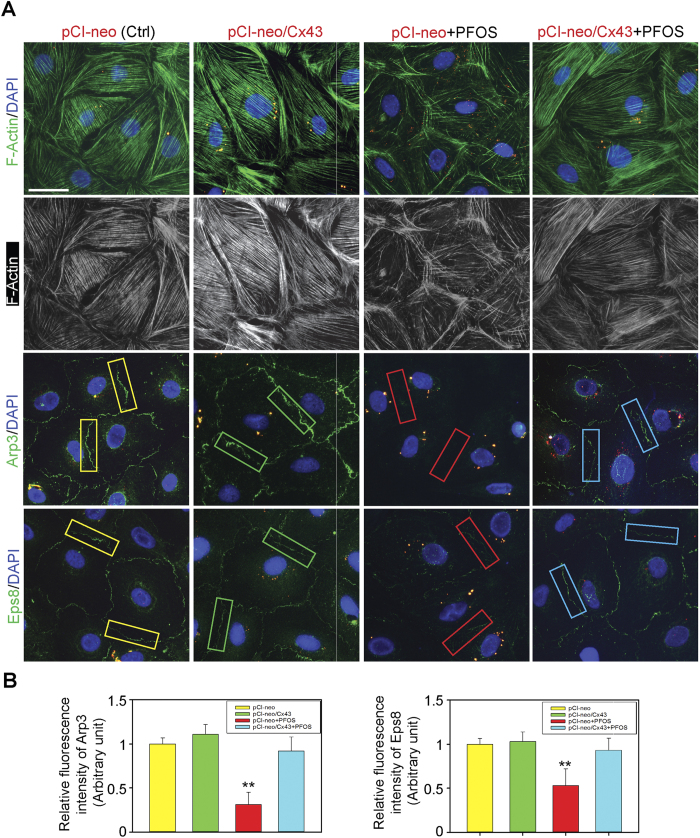
Overexpression of Cx43 in Sertoli cells restores actin microfilament organization via proper spatiotemporal expression of actin binding/regulatory proteins Arp3 and Eps8. (**A**) We next examined the mechanism by which Cx43 overexpression rescues the PFOS-induced Sertoli cell TJ-barrier disruption. Exposure of Sertoli cells with an established functional TJ-permeability barrier to PFOS for 24 hr was found to induce mis-orgranization of actin microfilaments wherein they displayed extensive defragmentation across the Sertoli cell cytosol. However, overexpression of Cx43 restored organization of actin microfilaments in Sertoli cells, apparently via proper spatiotemporal expression of actin binding and regulatory proteins Arp3 and Eps8, such that these proteins localized to the Sertoli cell-cell interface, similar to the control cells expressed with the empty pCI-neo vector. Plasmid DNA (pCI-neo vs. pCI-neo/Cx43) was labeled with Cy3 and appeared as red fluorescence to annotate successful transfection. Scale bar, 30 μm, which applies to all other micrographs. (**B**) Relative fluorescent intensity of Arp3 and Eps8 at the Sertoli cell-cell interface was quantified in the PFOS treatment group (pCI-neo+PFOS) *vs.* control (Ctrl, transfected with pCI-neo vector alone), Cx43 overexpression (pCI-neo/Cx43) and Cx43 overexpression plus PFOS (pCI-neo/Cx43+PFOS) groups. Each bar is a mean ± SD by taking the average fluorescent intensity from two opposite ends between adjacent Sertoli cells (see corresponding colored boxed area for Arp3 *vs.* Eps8 from different groups) from 50 randomly selected cells in each experiment with *n* = 3 independent experiments. **P < 0.01.

To better understand the molecular mechanism by which overexpression of Cx43 that rescues PFOS-induced F-actin dis-organization, we next examined the possible involvement of Arp3 and Eps8 (Fig. 5). Overexpression of Cx43 in Sertoli cells exposed to PFOS induced re-organization of actin microfilaments in which microfilaments were no longer found to be considerably truncated vs. PFOS treated cells without Cx43 overexpression (Fig. 5A), which thus supported proper localization of adhesion proteins as noted in Fig. 4. More important, this corrective organization of actin microfilaments in Sertoli cells following Cx43 overexpression in PFOS treated cells was mediated by proper spatial expression of barbed end actin nucleation protein Arp3 in Sertoli cells and also actin barbed end capping and bundling protein Eps8, making their pattern of localization analogous to control cells (Fig. 5A), and the corrective spatial localization and/or expression of these two actin regulatory proteins were summarized in Fig. 5B. These findings thus support the notion that Cx43 exerts its effects by conferring proper organization of actin microfilaments in Sertoli cells to rescue the PFOS-induced TJ-barrier dysfunction. It is noted that this reduced Arp3 *vs.* Eps8 expression at the Sertoli cell-cell interface as noted herein (Fig. 5A,B) represented a considerable shift in their distribution instead of a down-regulation in expression since these two actin binding/regulatory proteins appeared to be internalized and possibly distributed evenly across the Sertoli cell cytosol. This notion was indeed supported by no significant change in the overall expression of Arp3 or Eps8 after PFOS treatment (see Fig. 1B).

## Discussion

GJs are important cell junctions that confer cell-cell communication and are known to coordinate cellular events across a cell epithelium, such as the seminiferous epithelium during the epithelial cycle of spermatogenesis. For instance, studies have shown that GJs serve as the ultrastructure to transfer chemicals and/or signaling molecules up to ~1000 daltons between adjacent cells in an epithelium (for reviews, see refs 4,59 and 60). Studies by physiologists and toxicologists have also shown that GJs play a critical role in transcriptional regulation and/or toxicant-mediated epigenetic gene modifications since gap junctional intercellular communication (GJIC) is an important mechanism that integrate signaling pathways, which in turn control gene expression between epithelial cells of an epithelium (for reviews, see refs 61, 62, 63, 64). Thus, if a toxicant, such as PFOS, cadmium or BPA (bisphenol A), perturbs GJIC function in the Sertoli cell of the testis, multiple signaling pathways can be compromised. This might result in male reproductive dysfunction. Studies that define the molecular mechanisms by which PFOS mediates its toxicity in mammalian cells such as the Sertoli cell in the testis are important since PFOS, an environmental toxicant, is still commonly used in China and other developing countries because of its beneficial properties in consumer products such as serving as a stain repellent in clothing, carpets and draperies. However, its use in the U.S. and Canada has been banned in the late 2000s. Herein, PFOS was shown to block GJIC between Sertoli cells using a dye-transfer cell-cell communication assay based on FRAP^25^. Since studies have shown that Cx43 is an important GJ-based protein in the testis and studies have shown that it is also a target of environmental toxicants (for a review, see ref. 10). Furthermore, a recent report using adjudin-induced sterility via the use of an acute dose as a model, the phenotypes of the testis^19^,^20^ mimicked many of the disruptive changes in the testis found in Sertoli cell-specific Cx43 KO mice^11^,^13^. Interestingly, overexpression of Cx43 in the adjudin-treated testes was found to re-initiate spermatogenesis in particular re-booting meiosis since round spermatids were detected in a significant number of tubules^20^. However, spermiogenesis failed to occur since no elongating/elongated spermatids were detected in any of the tubules with signs of meiosis^20^. Nonetheless, studies using the adjudin model *vs.* the Sertoli cell-specific Cx43 KO genetic models^11^,^13^ have demonstrated the significance of Cx43-based GJ function since the presence of at least nine other GJs found in the testis^7^ failed to replace the lost function of Cx43. However, it is of interest to note that while treatment of Sertoli cell epithelium with PFOS reduces GJ-based communication function, the evidence that this partial uncoupling is responsible for the effects of the toxicant is corroborative in nature since PFOS is plausibly mediated its toxicity through multiple pathways. However, overexpression of Cx43 was found to re-establish the disrupted actin microfilament organization, reseal the Sertoli TJ-barrier, and re-distribute TJ and basal ES proteins to the Sertoli cell-cell interface. Moreover, these changes were mediated by proper spatiotemporal expression of Arp3 and Eps8. These findings thus support the notion that Cx43 disruption induced by PFOS is one of the major mechanisms underlying PFOS-induced Sertoli cell injury.

These earlier findings thus prompted us to examine the role of Cx43 in correcting PFOS-mediated Sertoli cell injury by overexpressing Cx43 in the Sertoli cells exposed to this environmental toxicant as reported herein. It was noted that successful overexpression of Cx43 in Sertoli cells would repair the disrupted GJIC between these cells. More important, this re-establishment of GJIC in the Sertoli cell epithelium *in vitro* with an established functional TJ-permeability barrier that mimics the Sertoli BTB *in vivo* is crucial to re-organize the Sertoli cell actin microfilaments, making their organization similar to control Sertoli cells in which long stretches of microfilaments lay across the cell cytosol instead of grossly truncated following PFOS exposure, thereby supporting the proper adhesion protein complex localization (e.g., occludin/ZO-1, N-cadherin/ß-catenin which utilize F-actin for their attachments) via corrective spatial expression of actin binding and regulatory proteins such as Arp3 and Eps8. These findings thus support the notion that the re-established GJ-based communication following Cx43 overexpression is used to maintain proper communications between Sertoli cells. This re-established GJ function in turn supports better organization of the cytoskeleton to maintain the Sertoli cell TJ-barrier function.

It is noted that Cx43 global KO leads to perinatal death due to defects in neural crest formation, vascular development, and heart malformation^65^,^66^,^67^,^68^. A recent report has shown that using mouse embryonic fibroblasts (MEFs) obtained from Cx43 knockout (KO) mice for an *in vitro* wound closure assay, these MEFs were found to display defects in cell polarity and directional cell movement due to defects in microtubule organizing center (MOC) as a result of Cx43 deficiency^69^. Furthermore, actin microfilaments at the wound edge of these MEFs also failed to properly align to confer directional cell movement, and these defects in actin- and microtubule-based cytoskeletal functions could be restored, at least in part, following overexpression of Cx43^69^. These findings thus support our observations that Cx43 mediates its effects through proper GJIC to re-establish the necessary signaling function to confer proper cytoskeletal organization. Work is now in progress to identify the downstream signaling function involved in these events.

In summary, we have demonstrated that PFOS exerts its toxic effects in the testis by disrupting the Cx43-based GJIC, which in turn perturbs the underlying actin-based cytoskeletal function. This thus affects cell adhesion protein complexes at the Sertoli cell-cell interface which utilize F-actin for their attachment, thereby disrupting the Sertoli cell TJ-permeability barrier function. However, these disruptive effects can be rescued via overexpression of Cx43 in PFOS-treated Sertoli cells. Further investigations should include studies to manipulate cytoskeletal function through their binding and regulatory proteins, such as Arp3, Eps8 and palladin for F-actin and EB1 and MARKs for MTs, attempting the block the PFOS-induced reproductive dysfunction.

## Additional Information

**How to cite this article**: Li, N. *et al*. Rescue of perfluorooctanesulfonate (PFOS)-mediated Sertoli cell injury by overexpression of gap junction protein connexin 43. *Sci. Rep.* **6**, 29667; doi: 10.1038/srep29667 (2016).

## Figures and Tables

**Table 1:** Antibodies used for different experiments in this report.

Antibodies	Host species	Vendor	Catalog number	Dilution	
				IB	IF
Actin	Goat	Santa Cruz Biotechnology	sc-1616	1:200	
Arp3	Mouse	Sigma-Aldrich	A5979	1:3000	1:100
α-catenin	Rabbit	Santa Cruz Biotechnology	sc-7894	1:200	
β-catenin	Mouse	Invitrogen	13-8400	1:500	1:100
β1-integrin	Rabbit	Santa Cruz Biotechnology	sc-8978	1:200	
Claudin 11	Rabbit	Invitrogen	36-4500	1:500	1:100
Cx43	Rabbit	Sigma-Aldrich	C6219	1:3000	1:100
Eps8	Mouse	BD Biosciences	610143	1:3000	1:100
N-cadherin	Mouse	Invitrogen	33-3900	1:500	1:100
N-WASP	Rabbit	Santa Cruz Biotechnology	sc-20770	1:200	
Occludin	Rabbit	Invitrogen	71-1500	1:250	
Vimentin	Mouse	Santa Cruz Biotechnology	sc-6260	1:1000	
ZO-1	Rabbit	Invitrogen	61-7300	1:500	1:100
